# Frosted Branch Angiitis Secondary to Familial Mediterranean Fever Resembling Central Retinal Vein Occlusion

**DOI:** 10.1155/2016/2916027

**Published:** 2016-12-04

**Authors:** Serdar Ozates, Pınar Çakar Ozdal, Mehmet Yasin Teke

**Affiliations:** Ankara Ulucanlar Eye Education and Research Hospital, Ankara, Turkey

## Abstract

*Purpose*. To report a case of unilateral frosted branch angiitis (FBA) resembling central retinal vein occlusion associated with Familial Mediterranean Fever (FMF).* Case Report*. A 32-year-old woman presented with progressive, painless vision loss in her left eye lasting for 2 days. She was clinically diagnosed with FMF 2 months ago. The best-corrected visual acuity (BCVA) was 20/20 in her right eye and there was light perception in the left. Ophthalmologic examination revealed severe retinal vasculitis showing clinical features of FBA in the left eye. 64 mg/day oral methylprednisolone was started. A significant improvement in retinal vasculitis was observed in two weeks. However, BCVA did not increase significantly due to subhyaloid premacular hemorrhage. Argon laser posterior hyaloidotomy was performed. One week after hyaloidotomy, visual acuity improved to 20/20 and intravitreal hemorrhage disappeared. Four months after the first attack, FBA recurred. Oral methylprednisolone dosage was increased to 64 mg/day and combined with azathioprine 150 mg. At the end of 12-month follow-up, the BCVA was 20/25 and development of epiretinal membrane was observed in the left eye.* Conclusions*. Frosted branch angiitis may occur with gene abnormalities as an underlying condition. Our case showed that FMF might be a causative disease.

## 1. Introduction

Frosted branch angiitis (FBA) is a rare vascular disorder, first reported by Ito et al. in 1976 in a six-year-old child [1]. Typically FBA is characterized by acute visual loss, severe vascular inflammation, continuous sheathing of vascular structures, and retinal hemorrhages. As Ito et al. described, FBA usually affects young and healthy persons [1]. In the upcoming years, several unusual FBA cases associated with ophthalmic and systemic diseases were reported. In the literature, other autoinflammatory diseases have a much higher incidence of ocular disease than Familial Mediterranean Fever (FMF) which was reported as a secondary cause of ophthalmic involvement in a few cases [2]. We present an unusual and rare case of FBA secondary to FMF resembling central retinal vein occlusion.

## 2. Case Report

**Figure 1:**
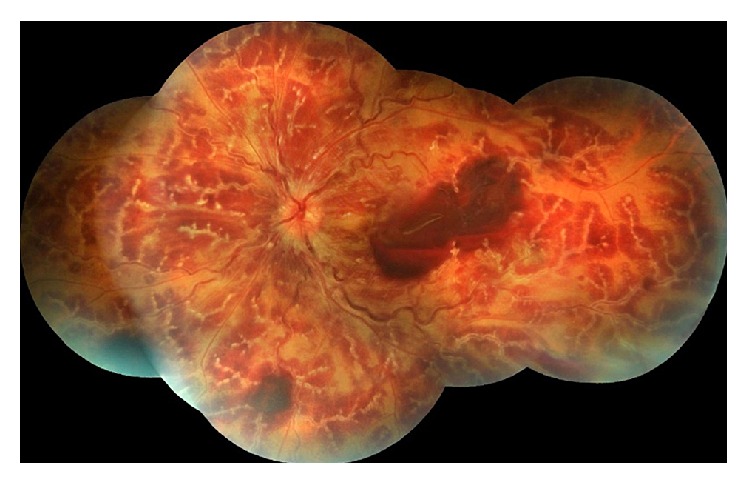
At presentation, left fundus image shows perivascular, white, and continuous sheathing of vascular structures starting from optic disc and extending to periphery. Papillary edema and subhyaloid hemorrhage in macular area were also observed.

A 32-year-old female presented to our hospital's emergency service with progressive, painless vision loss in her left eye lasting for 2 days. She was diagnosed with FMF based on her clinical complaints, familial history, and physical and laboratory examinations 2 months ago. She was started on oral colchicine therapy. She had no other history of systemic and ocular disease. Her best-corrected visual acuity (BCVA) was 20/20 in the right and there was light perception in the left eye, respectively. Intraocular pressure was in normal limits in both eyes. Slit lamp examination disclosed few cells in anterior chamber (+1) and anterior vitreous (+1) in the left eye. Fundus examination revealed perivascular, white, severe, and continuous sheathing of vascular structures starting from optic disc and extending to periphery. Veins were dilated and tortuous. Intraretinal hemorrhages in all quadrants, papillary edema, and subhyaloid hemorrhage in macular area were also observed (Figure 1). The ophthalmologic examination of the right eye was normal. Fluorescein angiography (FA) revealed dilated and tortuous veins and blockage of fluorescein due to retinal hemorrhages. Arteriovenous transit time was in normal limits. FA showed no venous stasis/occlusion and dye leakage at earlier phases. Later phases of FA could not be displayed because the patient had syncope and systemic hypotension at the second minute of FA.

Laboratory investigations, including complete blood count, electrolytes, plasma proteins, urea, creatinine, angiotensin converting enzyme, C-reactive protein, erythrocyte sedimentation rate (ESR), autoimmune markers (anti-cardiolipin antibodies, anti-neutrophil cytoplasmic antibodies, antinuclear antibody, antimitochondrial antibody, rheumatoid factor, anti-double-stranded DNA, anti-single-stranded DNA, anti-scl-70 antibodies, and anti-jo-1 antibodies), and antibody titers for HIV, CMV, HSV, EBV, syphilis, rubella, and toxoplasmosis were performed. Herpes simplex type 1 Ig G and rubella Ig G were positive. ESR was slightly high. Results of all other tests were in normal limits or negative. Cranial magnetic resonance imaging and chest X-ray were also normal. Genetic investigation revealed homozygous MEFV gene mutation (M964V) supporting FMF diagnosis. She had no clinical and laboratory signs of lymphoma, leukemia, sarcoidosis, tuberculosis, multiple sclerosis, systemic lupus erythematosus, Behçet's disease, and other autoimmune diseases.

**Figure 2:**
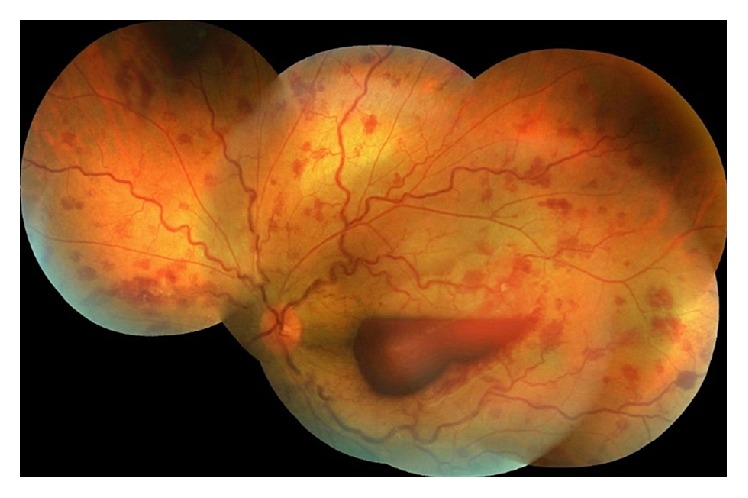
Visual acuity was poor because of subhyaloid premacular hemorrhage.

Systemic treatment of 64 mg/day oral methylprednisolone was started. Two days after the treatment, visual acuity of the left eye increased to 20/400. Cellular reaction of anterior chamber and vitreous disappeared. However, vascular sheathing, retinal hemorrhages, and subhyaloid premacular hemorrhage had no obvious change. One week after previous visit, fundus examination revealed significant improvement in the papillary edema, sheathing of vascular structures, and retinal hemorrhages. Visual acuity had no change due to subhyaloid premacular hemorrhage (Figure 2). Argon laser posterior hyaloidotomy was performed to the inferior part of the subhyaloid hemorrhage and the blood flowed through the hyaloidotomy to the vitreous cavity. Just after that the laser therapy visual acuity was 20/60. One week after hyaloidotomy, visual acuity significantly improved to 20/20 and dislocated intravitreal hemorrhage disappeared. Methylprednisolone was gradually tapered to 4 mg/day over 2 months.

**Figure 3:**
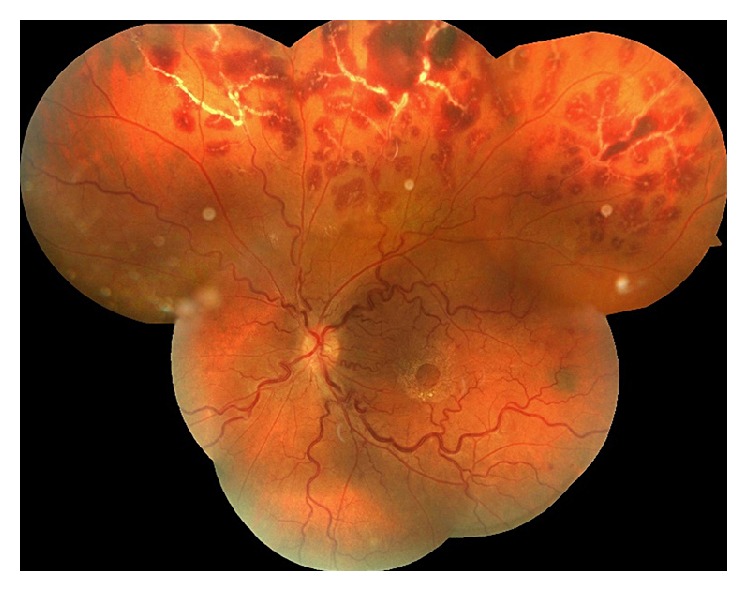
Recurrence of frosted branch angiitis. Fundus image shows vascular sheathing and preretinal and intraretinal hemorrhages limited to the superior peripheral retina.

Four months after first FBA attack, she presented to our retina department with a complaint of blurred vision for 2 days. Her BCVA was 20/20 in the right and 20/25 in the left eye, respectively. Biomicroscopy of anterior segment revealed anterior chamber (cells +1) and anterior vitreous inflammation (cells +1) in the left eye. Vascular sheathing and preretinal and intraretinal hemorrhages that were limited to superior peripheral retina were noted in the left eye (Figure 3). Oral methylprednisolone dosage was increased to 64 mg/day. Because the patient developed a “moon face” due to corticosteroid use and a recurrence of retinal vasculitis occurred, a decision for immunosuppressive therapy has been made. Corticosteroid treatment was combined with azathioprine 150 mg and methylprednisolone dosage was gradually tapered. Clinical findings of retinal vasculitis subsided over time and no other recurrences were noted during subsequent follow-ups. At her last visit performed in the 12th month of follow-up, the BCVA was 20/25 in the left eye. Biomicroscopy of anterior segment was normal. Fundus examination and optic coherence tomography showed epiretinal membrane formation around the fovea.

## 3. Discussion

Walker et al. stated that FBA usually affects young and healthy persons (especially females) and tends to be bilateral [3]. Consistently with previous studies, our patient was a 32-year-old female; however, FBA was unilateral in our case. Discussions about FBA being a new a clinical syndrome or an ocular sign of a secondary disease are still ongoing. Underlying risk factors for FBA and pathophysiology have not yet been clarified. FBA may be idiopathic or secondary to systemic disorders. Kleiner classified FBA into three subgroups based on possible secondary factors [4]. The first group is based on lymphoma and leukemia. Ocular manifestation of these malignancies may resemble FBA. In this situation, invasion and infiltration of vascular walls by malignant cells is the main mechanism in the pathophysiology. The second group is based on autoimmune diseases and infections. Inflammatory process like vasculitis, immune complex deposition, or hypersensitivity reactions to microorganisms is considered to be the underlying mechanism. The third group is referred to by “acute idiopathic frosted branch angiitis” and affects young, healthy persons. Our case can be considered in the second group of FBA, because FMF may trigger ocular vascular inflammation.

FMF is characterized by recurrent episodes of fever associated with polyserositis and arthritis [5]. Mutations in the* MEFV* gene which encodes pyrin protein are found associated with increased interleukin-1*β*, which causes uncontrolled inflammation [6]. Ocular manifestation in FMF is rare and only reported as cases. Michaelson et al. reported a case series of FMF patients with drusen-like colloid bodies at the level of Bruch membrane [7]. FMF cases with anterior uveitis, episkleritis, posterior episkleritis, and panuveitis were also reported in the literature [8–11]. It is still an unsolved problem as Yazici et al. highlighted that why ocular involvement is so rare in FMF compared to other autoinflammatory diseases which have ocular involvement in the majority of cases [9]. In the literature, Satoh et al. reported a case of FBA as a complication of FMF while on colchicine therapy [12]. In this patient final visual outcome was poor due to complicated CRVO. In our case, although clinical findings mimicked CRVO, FA showed no venous stasis/occlusion and elongation of transit time.

Clinical management of FBA in this case includes both systemic and ocular control of inflammation and preventing recurrences. The main treatment regime for FMF is colchicine therapy which prevents recurrences and reduces risk of amyloidosis [5]. However, colchicine was found less responsive to patients with homozygous MEFV gene mutation (M964V) [13]. This may be an explanation why FBA recurred while receiving 2 mg colchicine in our case. In the literature systemic corticosteroids are the main suggested treatment for FBA. Laboratory tests must be performed to exclude ocular-systemic infectious and treatable diseases like viral retinitis, toxoplasmosis, multiple sclerosis, and Behçet's disease, before using systemic corticosteroids. In our case, oral methylprednisolone was started as initial treatment. However, due to some side effects of corticosteroids, an antimetabolite agent azathioprine was started for steroid sparing purpose in long term.

Although several previously published studies reported favorable visual outcomes, sometimes prognosis may be poor due to retinal vein or artery occlusion, vitreous hemorrhage, epiretinal membrane, macular scarring, neovascular glaucoma, and optic atrophy as Kleiner reported [4]. In our case, although BCVA was 20/25 at the end of 12-month follow-up, an epiretinal membrane involving the macular area developed. To our knowledge this is the second case of FBA associated with FMF.

## 4. Conclusion

FBA could be ocular manifestation of secondary diseases and complicated cases may result in vision loss. Determination of underlying diseases and pathophysiology is the main concern for appropriate treatment. Although association between FBA and FMF is so rare, our case revealed that FMF should be considered as a causative disease in the differential diagnosis of FBA.
